# Danish general practitioners have found their own way of using point-of-care ultrasonography in primary care: a qualitative study

**DOI:** 10.1186/s12875-019-0984-x

**Published:** 2019-06-28

**Authors:** Camilla Aakjær Andersen, Annette Sofie Davidsen, John Brodersen, Ole Graumann, Martin Bach Jensen

**Affiliations:** 1 Center for General Practice at Aalborg University, Fyrkildevej 7, 1,13, 9220 Aalborg Øst, Denmark; 2 Research Unit for General Practice and Section of General Practice, Department of Public Health, Faculty of Health Sciences, University of Copenhagen, CSS, Øster Farimagsgade 5, DK-1014 Copenhagen, Denmark; 3 Centre of Research & Education in General Practice, Primary Health Care Research Unit, Region Zealand, Department of Public Health, Faculty of Health Sciences, University of Copenhagen, Øster Farimagsgade 5, P. O. Box 2099, DK-1014 Copenhagen, Denmark; 4 Department of Radiology, Radiological Innovation Unit, Odense University Hospital, J.B. Winsløws Vej 4, DK-5000 Odense, Denmark

**Keywords:** General practice, Family medicine, Primary care, Point-of-care ultrasound, Ultrasonography, Implementation, Qualitative methods, Interviews

## Abstract

**Background:**

General practitioners increasingly use point-of-care ultrasonography despite a lack of evidence-based guidelines for their appropriate use in primary care. Little is known about the integration of ultrasonography in general practice consultations and the impact of its use on patient care. The purpose of this study was to explore general practitioners’ experiences of using ultrasonography in the primary care setting.

**Methods:**

Adopting an explorative phenomenological approach, we performed semi-structured interviews with general practitioners who used ultrasonography in their daily work. Thirteen general practitioners were recruited stepwise, aiming for maximum variation in background characteristics. Interviews were conducted at the general practitioner’s own clinic. Transcription and systematic text condensation analysis began immediately after conducting each interview.

**Results:**

The general practitioners described using ultrasonography for both selected focused examinations and for explorative examinations. The two types of examinations were described differently for each of the following emerging themes: *motivation for using ultrasonography, ultrasonography as part of the consultation, selection of an ultrasound catalogue*, and *consequences of the general practitioner’s ultrasound examination*.

The general practitioners had chosen and integrated their own individual ultrasound catalogue of focused examinations as a natural part of their consultations. The focused examinations were used to answer simple clinical questions and they had a significant impact on the patients’ diagnoses, clinical pathways and treatments. The general practitioners considered their own catalogue of focused examinations as their comfort zone. However, they also performed explorative ultrasound examinations outside their catalogue. These scans were performed to train, gain or maintain ultrasound competences or as explorative examinations driven by curiosity. The explorative ultrasound examinations rarely had an impact on patient care.

**Conclusions:**

This study describes how general practitioners found their own way of using ultrasonography in general practice and selected a personal catalogue of ultrasound examinations that was applicable, relevant and meaningful for their daily clinical routines. This study may serve to inform implementation strategies in general practice by offering insights into central aspects that drive general practitioners’ behaviours.

**Electronic supplementary material:**

The online version of this article (10.1186/s12875-019-0984-x) contains supplementary material, which is available to authorized users.

## Background

The application of ultrasonography as an integrated part of clinical examinations has disseminated into many clinical specialities [1]. Today, ultrasound equipment is the size of a laptop, is affordable and improved resolution facilitates image interpretation. Moreover, the concept of point-of-care ultrasonography allows clinicians to focus on answering simple clinical questions e.g. ruling-in or out an abdominal aortic aneurism rather than providing a full description of an anatomical region [2].

Evidence from hospital settings supports clinicians’ use of ultrasonography as a bedside test providing earlier and more correct diagnosis and better subsequent treatment of patients [3, 4]. Some have argued that general practitioners (GPs) should also use frontline ultrasonography in their daily work [5–7]. However, there is very limited research informing GPs about which examinations to perform, how to integrate ultrasonography in general practice consultations and the overall advantages and disadvantages of using ultrasonography in general practice [8]. Evidence from the hospital setting is not necessarily transferable, as working conditions, patient populations and especially the epidemiology of symptoms of pathology is very different between the primary and the secondary sector. This challenges the opportunities for continuous supervision and the overall amount of ultrasound examinations, which is essential to gain or maintain competence. Moreover, GP workload is already high [9, 10] and adding ultrasonography may reduce GP accessibility for patients. Nevertheless, ultrasonography use has already spread; it is now used by GPs in several countries [8, 11], and the practice may disseminate further as GPs desire more point-of-care tests in general practice [12].

Attempts to guide the implementation of ultrasonography in general practice have been made [13]. In Denmark, a special interest group under the Danish College of General Practitioners (DSAM) developed a non-evidence-based consensus list of recommended ultrasound scans suited for GPs [14] (Additional file 1). However, knowledge dissemination regarding this list has been limited to a Facebook group [15], short reports on the DSAM homepage [16] and regional meetings for GPs with a special interest in ultrasonography. There are no official evidence-based guidelines on how to use ultrasonography in general practice, no educational programme for GPs or GPs in training, no description of what training is needed, and no registration of GPs using ultrasonography.

Previous implementation research in general practice has focused on exploring GP behaviours in relation to an externally imposed guideline or intervention [17]. By exploring the implementation of ultrasonography in general practice as an intervention introduced by GPs themselves, we may gain new insights into what motivates GPs towards changing their behaviours.

The use of ultrasonography among GPs in Denmark is limited to a minority of enthusiastic GPs [11] who navigate through uncharted waters. These GPs may possess valuable knowledge about how ultrasonography can be integrated and used in general practice consultations. Hence, the purpose of this study was to explore these GPs’ experiences in using ultrasonography in the primary care setting.

## Method

We used an explorative phenomenological approach [18] with individual semi-structured interviews to gain in-depth knowledge about the GPs’ experiences with ultrasonography.

All interviews were conducted, audio-recorded, transcribed verbatim and analysed by CAA, who is a medical doctor with clinical experience in general practice and of using ultrasonography in this setting. As CAA was very close to the field of interest, CAA’s assumptions and presuppositions were declared in a written document prior to commencing the study. Awareness about her presumptions made CAA more prepared to bracket these during the interviews.

### Setting

Denmark has a public healthcare system where almost all patients are listed with a GP for primary healthcare. The GP acts as a gatekeeper to secondary care. Treatment is financed by taxes and is free of charge for patients. GPs are paid by a mixture of capitation and fee-for-service reimbursement. GPs are free to select suitable services, but presently they receive no extra payment for performing ultrasound examinations, and expenses for ultrasound training and equipment must be covered by the GPs themselves.

### Participant selection

We recruited GPs (medical doctors with a full postgraduate specialization in family medicine) working in general practice from all over Denmark. Information about the study was distributed through ultrasonography teaching sessions, regional small-group learning meetings and the Danish GPs’ ultrasonography Facebook group [15]. Interested GPs were invited to provide their contact information and background characteristics through a small information form designed for this purpose (Additional file 2). Thirty-four GPs signed up to participate. From these 34 GPs, we purposely selected participants aiming for maximum variation in the following background characteristics: age, gender, experience as a GP, experience with ultrasonography, extent of chosen ultrasonography range, location in Denmark and organisational aspects of the practice. Recruitment was stepwise, aiming for 10–15 participants based on the concept of information power [19]. Recruitment stopped when no new knowledge emerged during the interviews.

### Interviews

The interviews were conducted at the GPs’ clinics to support and maintain their role as healthcare professionals in their everyday working environment. An interview guide (Additional file 3) was developed based on knowledge gained from a systematic literature review [8] and informal focus group discussions with GPs. The interview guide provided domains and suggested questions. However, the semi-structured nature of the interview meant that CAA could deviate from the structure and follow the interviewees’ narratives and any possibilities that arose. All participants provided their informed consent to participate. Transcriptions and audio recordings were anonymised according to the regulations of the Danish data protection agency.

### Data analysis

**Fig. 1:**
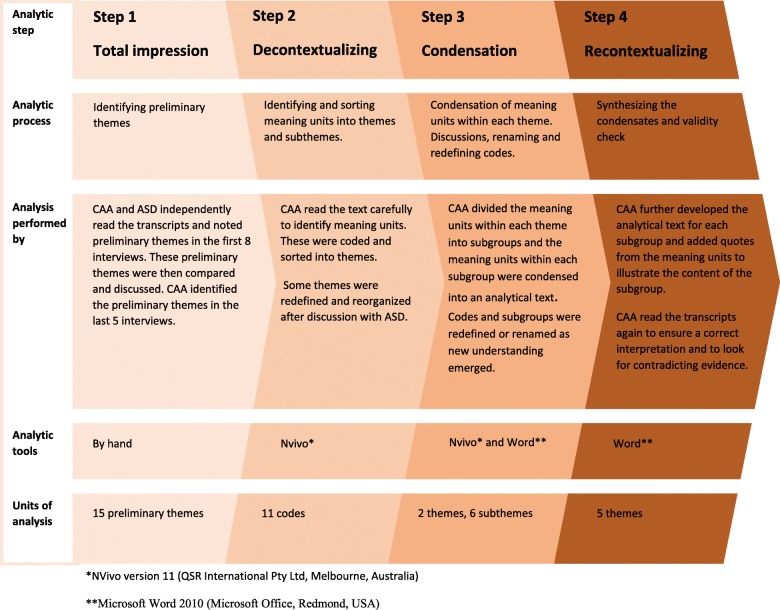
Systematic text condensation in this study. This figure illustrates and elaborates the analytic process in this study

We used systematic text condensation [20]. This is an inductive cross-case analysis consisting of four steps: total impression, identifying an sorting meaning units, condensation, and synthesizing (Fig. 1). Interviews and analysis were conducted in Danish. The analytical results and quotes were later translated to English in collaboration with a professional translator.

## Results

Thirteen GPs were included in the study between August 2016 and March 2017. Their characteristics are shown in Table 1. The GPs reflected a large variation in the use of ultrasonography regarding organs scanned, clinical conditions examined for and decisions made based on the ultrasound examination. The GPs had between a few months and more than 10 years of experience with ultrasonography, and all had participated in some ultrasound training. The extent of the training varied from supervised training during residency to extensive formalised education in line with specialists’ training.

**Table 1:** Characteristics of participants (*N* = 13)

Characteristic	No
Age	
40–50 years	6
51–60 years	4
61–70 years	3
Gender	
Male	11
Female	2
Experience as a general practitioner	
> 20 years	3
10–20 years	6
< 10 years	4
Experience using ultrasonography in general practice	
< 2 years	7
2–5 years	4
> 5 years	2
Extent of ultrasonography use	
Restricted to a few anatomical areas	5
According to the DSAM “common trunk”	5
Broad use	3
Frequency of ultrasonography use	
Several times a day (1–8)	4
Once a day	3
Several times a week	5
Several times a month	1
Practice location	
Urban	8
Mixed	5
North Denmark Region	2
Central Denmark Region	4
Region of Southern Denmark	2
Region Zealand	0
Capital Region of Denmark	5
Practice size	
< 2000 patients	4
2000–5000 patients	4
> 5000 patients	5
Type of practice	
Partnership practice	9
Solo-practice	2
Cooperation practice	2

**Fig. 2:**
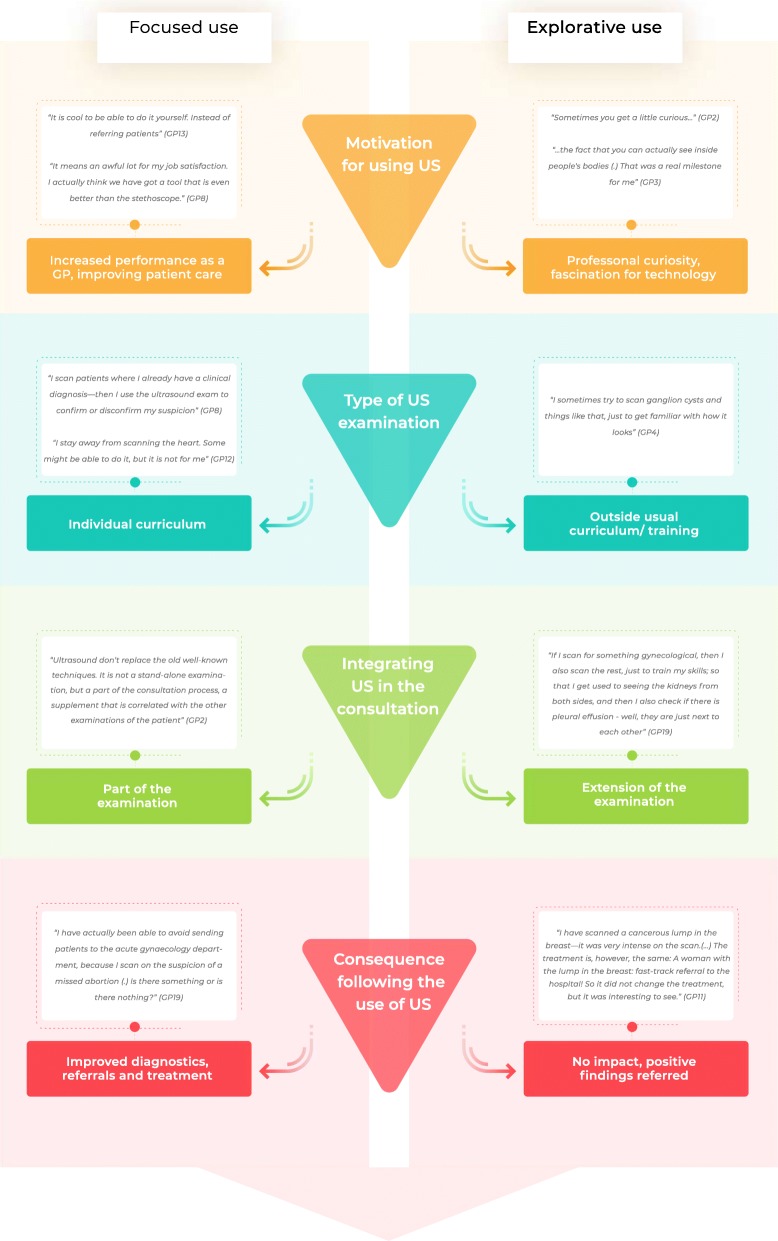
Two ways of using ultrasonography. This figure illustrates the interaction between themes in the analysis and the distinction between selected focused ultrasound examinations and explorative ultrasound examinations within each theme

Regardless of level of experience, all GPs described two ways of using ultrasonography in their daily practice: *selected focused ultrasound examinations* and *explorative ultrasound examinations*. The distinction between the two types was very clear among the novice GPs and was less pronounced among the most experienced GPs. Moreover, four themes were revealed*: motivation for using ultrasonography, ultrasonography as part of the consultation, selection of an ultrasound catalogue*, and *consequences of the GP ultrasound examination*. The characteristics of each type of examination and the four themes are elaborated in detail below and the interaction between themes is presented in Fig. 2.

### Two ways of using ultrasonography in general practice

#### Selected focused ultrasound examinations

All GPs described primarily using ultrasonography for selected point-of-care examinations to answer a clinical question raised by a patient’s history and/or by a physical examination of the patient. These selected examinations were limited to the description of specific organs and conditions within these organs. Hence, the examinations were performed only on symptomatic patients where the GPs aimed to confirm or disconfirm their clinical suspicions.

“I ask myself: Is this kidney big and has it distended due to hydronephrosis? If it turns out that there is a single bubble in the kidney’s parenchyma – well that’s not what I asked myself, so I don’t consider it” (GP15).

The GPs explained that these limited ultrasound examinations were different from traditional ultrasound examinations performed by radiologists.

“It is a clinical and not a diagnostic scan. To us, the ultrasound scan is just a tool. So I am not afraid to make – My scan isn’t a final document, unlike if I made something called a diagnostic scan.” (GP13).

Focused GP ultrasound examinations were restricted to rule-in pathology and not rule-out. However, some GPs said that they could rule out certain clinical conditions, e.g. spontaneous abortion, if they detected foetal heart movements in an intrauterine foetus.

When GPs performed focused ultrasound examinations, they worked within their comfort zone – their selected usual catalogue and they talked about the examinations with pride, providing several examples that underlined the importance of these examinations. They had experience in doing these examinations, felt sufficiently trained within this field and performed the examinations on a regular basis. They felt sure that they could perform the examinations and interpret the ultrasound images. Hence, they said that performing their focused ultrasound examinations provided them with a sense of reassurance.

#### Explorative ultrasound examinations

Although the GPs talked about the importance of knowing the extent and limits of their abilities and staying within their comfort zone, they all recounted that they sometimes deviated from their usual catalogue and performed more explorative ultrasound examinations. These extended examinations were performed either to train ultrasound competences or simply explore possible explanations for a patient’s symptoms without having a clear clinical suspicion of a specific disease.

Ultrasonography performed to train ultrasound competences was described by both novice and experienced GPs. The goal was to try new types of ultrasound examinations, to practice difficult ultrasound competences or simply train or maintain skills. These training examinations were often an extension of a focused examination of organs without clinical indication:

“If I scan for – let’s say gall stones – then I also locate the spleen and I check the kidneys and include the aorta, include the bladder…” (GP8).

Other ultrasound examinations were explorative by nature and mainly performed by GPs with more ultrasound experience. In these examinations, the GPs said that they did not have a clear focus, but they hoped they might find something that explained the patient’s symptoms. These examinations were clearly outside the GPs’ comfort zone and often not part of their previous ultrasound training.

“I sometimes extend my usual catalogue (.) when I think something is potentially interesting, but always with reservations uhm (…) I am (.) somewhat on shaky ground and I am prepared to say -if there is anything I am unsure about, then I refer it” (GP3).

The GPs talked about these examinations with some hesitation, underlining their explorative nature and insecurity. The GPs stated that they often aborted these examinations without reaching a conclusion.

### Motivation for using ultrasonography

The GPs described that they used ultrasonography for the sake of patients and society, but also out of self-interest. They felt that they improved patient care by increasing patients’ access to ultrasonography and by being able to complete patient treatment in general practice without referral. The GPs believed that they reduced the number of patients referred for further examination in the secondary healthcare sector, thereby reducing overall healthcare costs and at the same time providing better service for their patients. They also said that they could provide diagnostic certainty for patients and reduce their health concerns.

Most GPs claimed that their primary motivation for using ultrasonography was increased job enthusiasm and professional contentment. Ultrasonography gave them more satisfaction in their everyday work. They said that working as a GP had become more fun, exciting and varied after they had begun using ultrasonography. When they performed focused examinations, they felt that ultrasonography enabled them to perform better as a GP.

“Really, we are using it for our own satisfaction and to get better at diagnosing things. Being more certain about, for example, being able to guide an injection somewhere or things like that. It is a tool that helps us on a daily basis.” (GP4).

Explorative examinations gave the GPs’ professionalism a boost.

“I could just have done a normal exam - maybe there is a filling to the left, maybe not - and then send a referral – but now [after the ultrasound] I was able to send a referral where it is noted that I have found something measuring such and such and having more than one chamber, you know? That is – I think – really great.” (GP5).

The GPs said that they were fascinated by the technology and the possibility of seeing and diagnosing pathology that was previously out of reach. Some described feeling motivated by being first-movers, as they firmly believed that ultrasonography was part of the future in general practice.

### Ultrasonography as part of the consultation

The GPs described using ultrasonography as a natural continuum in consultations after hearing the patient’s history and conducting physical examinations. *Focused ultrasound examinations* were not seen as an outstanding examination, but rather as an integrated part of the diagnostic process – a supplement interpreted together with the patient’s history and the physical findings. Focused examinations were compared to other diagnostic tests in general practice, e.g. ECG, CRP or stethoscopy, and the GPs described how they integrated ultrasound in the same way:

“I see the scanner as an extension of my fingers; of course, I also examine people’s abdomen with my hands. In situations where I feel the edge of a liver below the curvature, I could use the scanner to explore if the liver extends [.] below the curvature. It [ultrasound] confirms what I am already doing” (GP15).

Explorative ultrasound scans were described as an additional examination that was performed after the initial diagnostic process and a conclusion had been reached. Indeed, some GPs described occasionally booking patients for a new consultation to have enough time to perform the ultrasound examination. Some GPs described how the focused examinations were conducted within the timeframe of the consultation, while others described that the duration of the consultation was extended with 5–10 min, which sometimes made them fall behind schedule.

### Selection of an individual ultrasound catalogue

#### Individual selection

All the GPs said that they had selected an individual ultrasound catalogue of focused examinations based on their patient population, professional interests and ultrasound training. The individual catalogue included ultrasound examinations that were meaningful for the GP. Most of the GPs had limited their catalogue to simple clinical conditions within a few anatomical areas, while more experienced GPs had a larger catalogue.

“We have also limited ourselves to some selected musculoskeletal joints. Clearly, we can’t scan all joints, so we have limited ourselves to specific joints – partly to diagnose and also for giving ultrasound-guided injections” (GP13).

None of the GPs expressed full commitment to the DSAM list of recommended ultrasound scans for GPs. Many were not aware of this list and the remaining GPs said that they had either chosen some of the examinations from the list or developed a personal catalogue that exceeded the list.

Most of the GPs had an ultrasound training that was more extensive than was reflected in their individual catalogue, and all described having excluded some ultrasound examinations because they were considered “out of reach” or without any impact for patients. Out of reach examinations were described as being too difficult to perform, too time-consuming, too rare in general practice or examinations that the GP felt insufficiently trained to conduct. Ultrasound examinations without impact for patient care were described as examinations where the diagnosis was primarily clinical, e.g. a tennis elbow, or examinations where a GP ultrasound examination could not replace examination by an ultrasound specialist, e.g. a radiologist. Generally, GPs were reluctant to scan children, and to scan on patients’ requests. Some ultrasound scans, e.g. screening of the abdominal aorta, life-threatening conditions, and cancer-related scans, were excluded because the GPs felt that they were ill-suited for general practice.

#### General practitioners are not radiologists

GPs described that using ultrasonography required education, practice, dedication and technical and anatomical understanding, but most importantly humility and respect. Ultrasonography was described as a user-dependent technology, and GPs emphasized that they were generalists and not radiologists. They were not trying to do a radiologist’s job. Indeed, they described their use of ultrasonography as being limited to examinations on a different level.

“After all, there is a limit as to how skilled we can become as we need to do other things, too; so of course, you can’t reach the same level as the others (radiologists), you can’t, and I don’t think that’s the point, so I – it’s more to get by.” (GP14).

The GPs described how they – as generalists – were used to performing at a certain level before referring patients to specialists and that knowing one’s limitations was of paramount importance.

“In principle, you can scan everything, but you can’t be good at everything.” (GP3).

Some of the GPs stated that radiologists could rule out pathology using ultrasound examinations, while that would never be the case with the limited examinations in general practice. It was considered important and necessary to communicate about the differences between ultrasonography performed by a radiologist and a GP both to patients and specialists in the secondary healthcare sector to avoid false expectations.

### Consequences of the GP ultrasound examination

The GPs described ultrasonography as having a big impact on their diagnostic processes. They felt more confident in their diagnosis after using ultrasound compared to the traditional examination of patients, which they felt entailed considerable uncertainties.

“You’re a little more confident that (.) what you are doing is correct in terms of not risking mistreating patients” (GP14).

The GPs recounted that focused ultrasound examinations led to faster and more precise diagnosis and treatment either in general practice or through more qualified referrals to the secondary sector.

“I make better and higher quality diagnoses or referrals” (GP5).

Two of the GPs argued that ultrasonography use in general practice was merely a service improvement rather than an actual improvement in patient care. Overall, patients were treated in the same way as before, but diagnoses were reached faster, and more treatments were finalised in primary care.

When the GPs carried out ultrasonography outside their usual individual catalogue, they were aware that they were doing this for their own benefit. Hence, they were very cautious about drawing conclusions and taking consequences. Often, the plan for further action was made beforehand, and ultrasonography only influenced this in case of positive incidental findings. Several GPs gave examples of cases with incidental findings. In all these examples, the incidental findings were considered to relate to a serious disease, e.g. cancer, and the patient’s clinical pathway was accelerated through fast-track referrals. However, some incidental findings turned out to be false alarms. Generally, more experienced GPs felt more confident in their ultrasound findings than novices did, and they described their ultrasound examinations as having a greater impact on patient care. Novice GPs were very careful about ruling out pathology. They still relied on their clinical examinations and mostly used ultrasonography to confirm their clinical findings.

All GPs described situations where they felt unable to interpret ultrasound images and had to use their usual treatments and pathways. This often entailed referring patients for an ultrasound examination in the secondary healthcare sector.

“Some patients are difficult to scan and some have organs that are placed where you wouldn’t expect. In those cases, you can’t answer clinical questions with ultrasound, and so these patients receive the treatment they would otherwise have received.” (GP15).

The GPs expressed little concern about the risks of their own use of ultrasonography. They said that misdiagnosis was only made by GPs with insufficient training, poor judgment of their own ultrasound skills or too much use of ultrasonography for explorative purposes. The GPs in this study were not concerned about overlooking pathology in their limited scans as they were reluctant to rule out pathology based exclusively on the scans. Overdiagnosis was described as a general concern in the healthcare system and not specifically related to their use of ultrasonography.

The GPs explained that they felt that ultrasonography improved the doctor-patient relationship through better dialogue and a more positive atmosphere in consultations. However, some GPs described that ultrasonography gave them authority and increased the patient’s confidence and respect for the doctor.

“The doctor becomes more appreciated because he is more thorough, as he has this extra magical examination, which is almost worse than the stethoscope. You know, it’s crazy, now we can actually see inside the body! That’s the kind of magic it has.” (GP19).

The GPs experienced that the patients became reassured by ultrasonography and generally, the GPs thought that patients appreciated technology in GP consultations. However, some GPs expressed concern about patients’ reliance on the technology. Although the GPs tried to explain these limitations, they felt unsure, whether patients were able to understand these limitations or differentiate between a specialist ultrasound examination and a GP ultrasound examination.

## Discussion

To our knowledge, this study is the first to provide an in-depth understanding of GPs’ use of ultrasonography in the primary healthcare setting. We found that GPs in Denmark use ultrasonography in two different ways: selected focused ultrasound examinations and explorative ultrasound examinations. The former were selected scans within each GP’s own individually chosen ultrasound catalogue. These examinations were believed to have a high impact on patient care and provide reassurance for both the GP and the patient. The GPs spoke with pride about these examinations and gave several examples where their thought their use of these scans had improved patients’ outcomes. However, the GPs did not restrict themselves to performing only selected focused examinations. They also described performing explorative ultrasound scans that were beyond their individual standard catalogue. These examinations were performed to various degrees to either practise ultrasound skills or find explanations for a patient’s symptoms. The GPs talked about explorative ultrasound examinations with some hesitation and underlined that they performed these scans with caution. According to the GPs, explorative scans rarely had an impact on patient care, but some GPs described how incidental findings had resulted in referral for further examination due to suspicion of a serious disease. The GPs had few concerns about their own use of ultrasonography. They believed they were improving patient care and that they were first-movers with this technology, which entailed learning by doing.

### Strengths and limitations

We took several precautions to secure the confirmability of this study: The coding and analytical process was supervised by a senior qualitative researcher (ASD) with no experience in ultrasonography and the interview transcripts were re-read thoroughly, looking for contradictory evidence of the distinction between the two types of ultrasound examinations described in Fig. 2. Furthermore, the results were compared to CAA’s written assumptions and presuppositions to secure the production of new knowledge.

We aimed for maximum variation in our sample. However, the selection of GPs was limited to those responding to our invitation. Only a few women and no GPs from rural practices responded. This may have limited the credibility of our study.

The transferability of our results may be compromised as our study only included GPs in general practice in Denmark. The organisational aspects of the Danish healthcare system such as workload in primary care, lack of reimbursement for conducting ultrasonography and availability of this technique for patients may influence GPs’ ways of using ultrasonography. However, these organisational aspects have also been described in other countries [21, 22]. Hence, our results are likely to extend to GPs in other countries.

### Findings in context

The GPs in this study described their use of ultrasonography as something distinct from the traditional ultrasound examination performed by radiologists in the secondary healthcare sector - not as an inferior examination, but as a separate examination. They described the need for both types of examinations, that they were not trying to be radiologists and that some ultrasound examinations should still be undertaken by specialists. This confirms a theory proposed by Weile et al. [2] that point-of-care ultrasound performed by a clinician represents a case of disruptive innovation. They argue that point-of-care ultrasound has a different trajectory than the traditional full ultrasound examination with different strengths, limitations, opportunities and patients. This understanding may explain why the GPs in the present study expressed little concern about their own use of the technology.

The GPs were highly motivated and had implemented ultrasonography in their daily clinical routines with no organisational guidance or financial incentives. They had found their own way and developed an individual catalogue of ultrasound examinations that were applicable, relevant and meaningful to them. This change in practice – including a selection of ultrasound examinations in daily routines – may be understood with respect to the COM-B model introduced by Michie [23]. The model describes *Capability, Opportunity and Motivation* as central components that generate *Behaviour* and form the Behaviour Change Wheel. When GPs performed their selected ultrasound examinations, all three components were met: the GPs felt that they possessed the necessary skills and knowledge (Capability); the ultrasound examinations were integrated into the patient examination, conducted within the timeframe of the consultation and the GPs had the equipment to perform the examination (Opportunity). Finally, the GPs were highly motivated to perform the examinations based on previous experiences. They believed that they performed better as GPs, improved patient care and felt increased professional satisfaction (Motivation). Moreover, some ultrasound examinations were de-selected by the GPs because they felt insufficiently trained or the examination was too difficult to perform (lack of Capability), they did not have the necessary probes for the examination or time to perform the examination (lack of Opportunity), or they felt that the examination did not affect patient care (lack of Motivation).

Although the GPs’ educational levels differed considerably, they all described occasionally performing ultrasound examinations to gain, train or maintain their skills. Novice GPs primarily used ultrasonography for focused examinations, while more experienced GPs also performed explorative scans without a specific clinical question. This difference could be explained by the GPs’ different competence levels. Dreyfys and Dreyfys [24] describe professionals moving through five stages from novice to expert. Rules and analytical reasoning dominate the first steps of the learning process, while later stages are dominated by intuitive use based on pattern recognition. In our study, the more experienced GPs described a more intuitive use of ultrasonography with a less clear distinction between focused and explorative use, while novice GPs predominantly stayed within their comfort zone and described a more rigorous use. Carraccio et al. [25] describe that learners require exposure to environments and clinical scenarios outside their normal comfort zone in order to stimulate their adaptive higher-level clinical reasoning that is critical for transforming proficient practitioners into expert practitioners. Hence, the explorative examinations described by the GPs in the present study may be seen as an inevitable part of a learning process, although lack of opportunity and capability also limit the extent of this process.

We found that GPs use ultrasonography for different organs and with different indications. This is in line with the findings in a previous literature review [8]. Radiologists and other specialties have raised the question of whether GPs as generalists can carry out ultrasound examinations within all areas, and have expressed a need for organisational restrictions [1, 26, 27]. However, previous research has shown that GPs do not necessarily follow guidelines [28]. The GPs in our study had made their own restrictions and selected their own curricula of ultrasound examination based on experience. All GPs in the present study had participated in ultrasound training, but their catalogues were determined by their individual experiences and they did not stay within their comfort zone. Although some of the GPs were aware of the recommended list of scans for GPs – the DSAM “common trunk” – none of the GPs scanned solely according to this list. Since previous studies have shown that some ultrasound examinations may be more difficult to learn, more time-consuming and carry a higher risk of causing harm to patients [8], restrictions may serve to guide the GPs in appropriate use. However, too many restrictions may limit the development of skills and competences.

This study describes how the lack of evidence-based guidelines for education and use of ultrasonography by GPs makes ultrasonography in general practice any individual’s game. GPs are left to navigate themselves through choosing equipment, selecting the right training, making sure to maintain skills and selecting which examinations to perform on patients. Some of the GPs described how ultrasonography increased the consultation time and this might increase GP workload and affect accessibility for patients. These findings may extend beyond general practice and apply to other clinicians and implementation of other new technologies.

Recently, an American guideline has been developed for ultrasound training of family medicine residents [13]. This guideline is based primarily on evidence from a hospital setting and is very comprehensive, and hence not necessarily applicable for GPs working in primary care. To be implemented and to avoid spectrum bias, an evidence-based guideline for GP ultrasound examinations must reflect the patient population and the working conditions in the relevant general practice. Future research should investigate which ultrasound examinations are relevant in the general practice setting and can be performed with adequate diagnostic precision, how ultrasound competence is reached and maintained, and how the use of ultrasonography in general practice affects patient care in the long term.

## Conclusion

This study describes how GPs have found their own way of using ultrasonography in primary care. The GPs were highly motivated to use ultrasound and described increased job enthusiasm and professional satisfaction. They selected a personal catalogue of focused ultrasound examinations that were applicable, relevant and meaningful in their daily clinical routines and had an impact on patient care. However, they also scanned outside this catalogue to train ultrasound competences, gain new competences and explore possible explanations for a patient’s symptoms. When the GPs extended their usual ultrasound catalogue, they were motivated by curiosity and a fascination for the technology. This study may serve to inform an implementation strategy for ultrasonography and other technological innovations in general practice by offering insights into central aspects that drive GPs’ behaviour.

## Additional files

Additional file 1: DSAM common trunk. This additional file provided the recommended list of ultrasound examinations suitable for general practice as suggested by the Danish ultrasound society for general practice. (PDF 455 kb)

Additional file 2: Collected background information. This additional file describes the collected background information use in the recruitment of participant for this study. (PDF 394 kb)

Additional file 3: Interview guide. This additional file provides the interview guide used in the study. (PDF 555 kb)

## Data Availability

The anonymized transcriptions and analytic datasets are available stored at Center for General practice at Aalborg University, Denmark according to regulations by the Danish Data Protection Agency. Anonymized data are available on request by contacting the corresponding author.

## Abbreviations

CRP: C-reactive protein
DSAM: The Danish College of General Practitioners
ECG: Electro cardiogram
GP: General Practitioner
