# Supplementary material for: Danish general practitioners have found their own way of using point-of-care ultrasonography in primary care: a qualitative study
Source: BMC Fam Pract. 2019 Jun 28;20:89. doi: 10.1186/s12875-019-0984-x (PMC6599254; doi:10.1186/s12875-019-0984-x)
Supplement: Supplementary file 1 — DSAM common trunk. This additional file provided the recommended list of ultrasound examinations suitable for general practice as suggested by the Danish ultrasound society for general practice. (PDF 455 kb) [file 12875_2019_984_MOESM1_ESM.pdf]

Additional file 1: DSAM common trunk

Danish general practitioners have found their own way of using point-of-care ultrasonography in primary care: A qualitative study

This additional file contains a translation of a list of recommended point-of-care ultrasound examinations suitable for general practitioners working in primary care. This consensus list was developed in 2016 by an interest group under The Danish College of General Practitioners (DSAM) in collaboration with radiologists. The list is based on clinical questions, that can be answered using point-of-care ultrasound.

The list was published in Danish in 2016 and is available at: https://www.dsam.dk/flx/organisation/udvalg_og_interessegrupper/ultralyd_i_almen_praksis/common_trunk/

The translation of the list was made by the principal author of this study.

DSAM common trunk

Focused ultrasound of the heart (FATE protocol)

Reduced contractility? (eyeball method)
Chamber size and shape?
Myocardial wall thickness?
Pericardial effusion?
Pleural effusion?

Focused Lung ultrasound (LUS protocol)

Interstitial syndrome?
Lung consolidation?
Pneumothorax?
Pleural effusion?

Focused ultrasound of the abdomen:

Ascites? (FAST protocol)
Abdominal aortic aneurism?
Gallbladder stones?
Cholecystitis? (Sonographic Murphy sign, gallbladder wall thickening (>3 mm), pericholecystic fluid)
Hydronephrosis?
Post-void residual urine?

Focused gynecological ultrasound:

Intrauterine fetus?
Crown-rump length (CRL)
Fetal heartbeat?
Intrauterine device (IUD)?

Focused ultrasound of the shoulder

Rupture of the long head of biceps tendon? (empty bicipital groove)

Fluid surrounding the biceps tendon? (joint pathology or tenosynovitis)
Subacromial bursitis?

Focused ultrasound of the elbow

Lateral epicondylitis (Tennis elbow)?

Focused ultrasound of the knee

Knee effusion? (suprapatellar pouch recesses)
Patellar tendinopathy? (jumper's knee)
Osgood-Schlatter?
Baker’s cyst?

Focused ultrasound of the foot

Achilles tendon tear?
Achilles tendinopathy?
Plantar fasciitis?

Ultrasound for Deep Venous Thrombosis detection

2-point compression ultrasound (common femoral vein and popliteal vein compression only)

Visual thrombus?
Flow?

Ultrasound of subcutaneous masses

Abscess or phlegmon?

Ultrasound for interventions

Venous access (e.g. blood samples)
Musculoskeletal injections
