# Supplementary material for: Danish general practitioners have found their own way of using point-of-care ultrasonography in primary care: a qualitative study
Source: BMC Fam Pract. 2019 Jun 28;20:89. doi: 10.1186/s12875-019-0984-x (PMC6599254; doi:10.1186/s12875-019-0984-x)
Supplement: Supplementary file 2 — Collected background information. This additional file describes the collected background information use in the recruitment of participant for this study. (PDF 394 kb) [file 12875_2019_984_MOESM2_ESM.pdf]

Additional file 2: Collected background information

Danish general practitioners have found their own way of using point-of-care ultrasonography in primary care: A qualitative study

We used the following questions in the recruitment process:

How long have you been using ultrasound in general practice?

Which type of ultrasound examinations do you perform in general practice?

Do you have colleagues in the clinic who use ultrasound?

How many doctors are working in your clinic?

Contact information provided information about clinic location.
