# Supplementary material for: Danish general practitioners have found their own way of using point-of-care ultrasonography in primary care: a qualitative study
Source: BMC Fam Pract. 2019 Jun 28;20:89. doi: 10.1186/s12875-019-0984-x (PMC6599254; doi:10.1186/s12875-019-0984-x)
Supplement: Supplementary file 3 — Interview guide. This additional file provides the interview guide used in the study. (PDF 555 kb) [file 12875_2019_984_MOESM3_ESM.pdf]

Additional file 3: Interview guide

Danish general practitioners have found their own way of using point-of-care ultrasonography in primary care: A qualitative study

Domain	Subdomain	Question
incentive	incentive	What made you interested in ultrasound?<br>What inspired you?
	Normalization	What made you choose to integrate ultrasound in your daily work as a GP?
	Introduction	What were you using ultrasound for in the beginning?
Use	Expansion	So what do you use ultrasound for today?
	Frequency	How often do you use ultrasound a day?
	Limitations	Are there some patients you do not scan? E.g. children
	Limitations	Are there some ultrasound examinations you do not perform? Why not?
Experience	Experience	How did you learn to use ultrasound?
	Training	Have you participated in a course or some sort of formalized training?
	Training	Could you elaborate on the content of this training?
	Experience	How long have you been using ultrasound?
	Supervision	Some doctors participate in groups for continuous learning. Do you participate in such a group concerning ultrasound in general practice?
Use	Indication	Could you describe for me, a typical situation in your daily clinic where you decide to use ultrasound?
	Indication	Why do you typically use ultrasound? Is it because you have a clinical situation where you are in doubt?
	screening	Do you sometimes scan more than you set out to scan?

	documentation	How do you document you findings?
Communication	Information to the patient	How do you inform the patient before, during and after the ultrasound examination?
	Expectations	Is it easy to inform the patients them and explain the examination?
	Expectations	Do you experience the patients have certain expectations to the ultrasound examination or to you as the deliver?
Consequences	Diagnostics	How big an influence does ultrasound have in your diagnostic process?
	Diagnostics	Could you give me some examples where ultrasound made a difference?
	Diagnostics	Do you have examples where ultrasound did not make the same difference?
Use	Diagnostics	Do you use ultrasound as a rule-in test or a rule-out test?
	Diagnostics	Do you always feel conficent in your ultrasound findings?
	Diagnostics	Are you able to rule-out disease based on your ultrasound examination?
	Diagnostics	What do you do when you fell uncertain?
Consequences	consultation	In your experience, how does ultrasound affect the consultation in general practice?
	consultation	How does ultrasound affect the doctor-patient relationship?
	consultation	Have yoy experienced that ultrasound makes you focus less on other elements of the consultaion?
Use	Clinical pathway	Could to give me examples of what you do when you have a positive finding on your ultrasound examination? Are there other situations where you would do something else?
Consequences	Clinical pathway	Could you give me examples where ultrasound have made a positive difference in the clinical patient pathway?
		Could you give me examples where ultrasound have made

		a negative difference in the clinical patient pathway?
